# P-236. The Effect of Hospitalization on Neurocognitive Function in People with HIV

**DOI:** 10.1093/ofid/ofaf695.458

**Published:** 2026-01-11

**Authors:** Lakshmi Chauhan, Kunling Wu, Frank J Palella, Todd T Brown, Leah H Rubin, Alison G Abraham, Ronnie Kasirye, Susan L Koletar, Katherine Tassiopoulos, Kristine M Erlandson

**Affiliations:** University of Colorado, Aurora, CO; Harvard University T H Chan School of Public Health, Boston, Massachusetts; Division of Infectious Diseases, Northwestern University, Feinberg School of Medicine, Chicago, Illinois, Chicago, IL; Johns Hopkins, Baltimore, Maryland; Johns Hopkins University School of Medicine, Baltimore, Maryland; University of Colorado, Aurora, CO; MU-JHU, Kampala, Kampala, Uganda; Ohio State University, Columbus, Ohio; Harvard University T H Chan School of Public Health, Boston, Massachusetts; University of Colorado Anschutz Medical Campus, Aurora, CO

## Abstract

**Background:**

Hospitalization contributes to cognitive decline among older adults without HIV; people with HIV (PWH) may have greater vulnerability for post-hospitalization decline. We aimed to determine the impact of hospitalization on trajectories of neurocognitive function in the long-term observational ACTG HAILO study.

**Figure:**
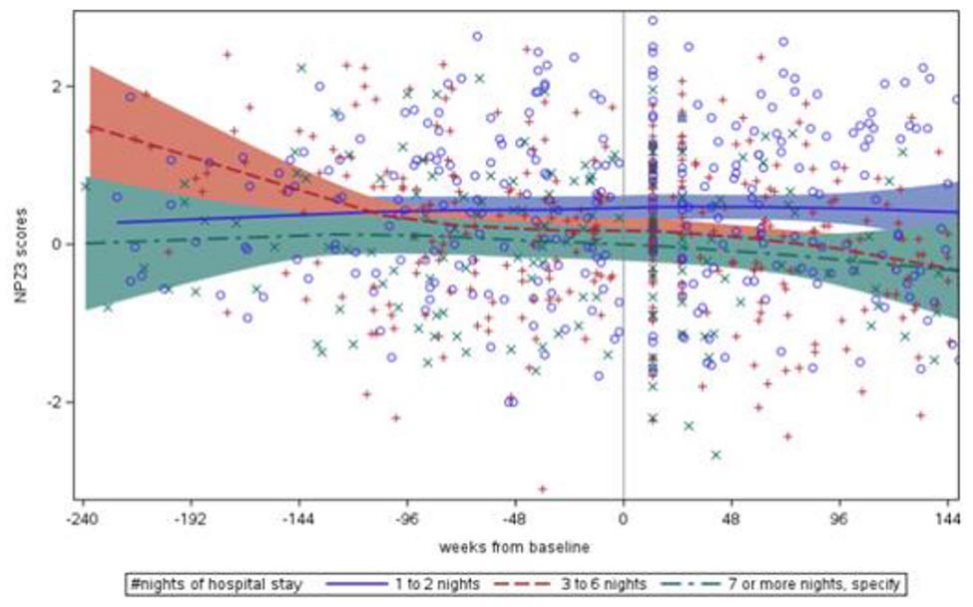
Neurocognitive scores (NPZ3 scores) before and after hospitalization

Demographics and variables at baseline among participants who were hospitalized and had NPZ3 data (N=199)

**Figure:**
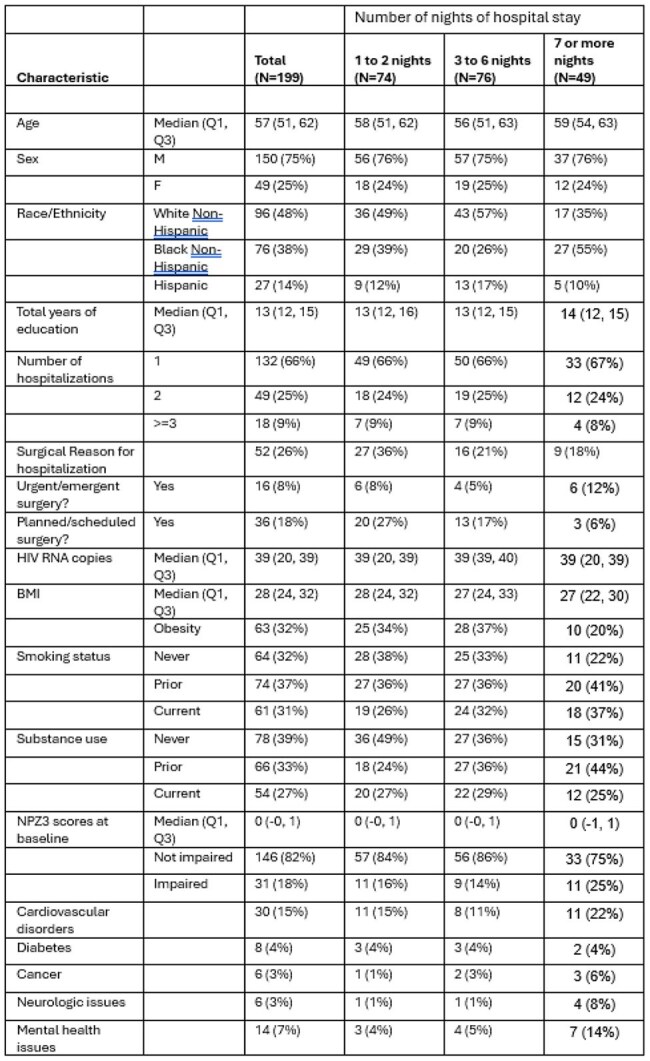

**Methods:**

We included participants who required hospitalization and had neurocognitive assessments before and up to 3 years after hospitalization. Demographics, self-reported information for first hospitalization (baseline) within the prior 6-month period were obtained. Outcomes included an average of 3 normalized z-scores of Trail-Making A and B and Digit symbol (NPZ3), with impairment defined as NPZ3 ≤-2 on 1 normalized z-score or ≤-1 on 2 normalized z-scores and change of 0.5 considered clinically relevant. Potential confounding variables (age, race/ethnicity, education, smoking, BMI, substance use, CD4 and HIV RNA viral load) were included in adjusted models if slope changed by ≥10%.

**Results:**

Of 892 participants, 208 participants had at least 1 hospitalization; 199 had NPZ3 scores before/after hospitalization. The median age was 57 (IQR 51, 62), 38% Black, and 14% Hispanic; median CD4 was 662 cells/mm3 (IQR 450,818), 94% had HIV-1 < 200 copies/ml. 74% were admitted for non-surgical reasons (Table 1). Baseline NPZ3 scores were impaired in 16% admitted for 1-2 days, 14% for 3-6 days and 25% for ≥ 7 days. Adjusting for race/ethnicity, the overall slope of NPZ3 scores before and after hospitalization was -0.103[-0.182, -0.024], p=0.01 (Figure 1), with the greatest change in those admitted for 3-6 days: 0.181[-0.307, -0.054], p= 0.005. Hospitalization for non-surgical reasons was associated with an NPZ3 slope decline of -0.117[-0.212,-0.022], p=0.02 vs surgical reasons -0.071[-0.206, 0.065], p=0.2.

**Conclusion:**

Small but persistent declines in neurocognitive scores were observed following hospitalization, notably in those admitted for 3-6 days and for non-surgical reasons. Monitoring for decline of neurocognitive function post hospitalization could help initiate earlier interventions.

**Disclosures:**

Frank J. Palella, MD, EMD Serono: Honoraria|Gilead Sciences: Honoraria|Merck: Honoraria|ViiV: Honoraria Todd T. Brown, MD, PhD, EMD Serono: Advisor/Consultant|GSK: Advisor/Consultant|Merck: Advisor/Consultant|ViiV: Advisor/Consultant Susan L. Koletar, MD, Gilead Sciences: Co-investigator on several grants; no direct salary support|ViiV Healthcare: Grant/Research Support Kristine M. Erlandson, MD MS, Gilead: Advisor/Consultant|Gilead: Grant/Research Support|ViiV: Advisor/Consultant|ViiV: Travel to meeting

